# Supplementary material for: Synthetically-primed adaptation of Pseudomonas putida to a non-native substrate D-xylose
Source: Nat Commun. 2024 Mar 26;15:2666. doi: 10.1038/s41467-024-46812-9 (PMC10965963; doi:10.1038/s41467-024-46812-9)
Supplement: Supplementary file 1 — Supplementary Information [file 41467_2024_46812_MOESM1_ESM.pdf]

Synthetically-primed adaptation of *Pseudomonas putida* to a non-native substrate D-xylose

Dvořák *et al.*

Supplementary Method 1. Enzyme assays

For enzyme activity measurements in *P. putida* EM42 strains, cell lysates were prepared by lysing cells cultured in 50 mL of LB medium with kanamycin or 50 mL of M9 medium with 2 g L^{-1} xylose or glucose and kanamycin. These cultures were inoculated to a starting OD_{600} of 0.05 from night cultures (grown for 16 h) in 10 mL of LB medium with kanamycin and grown while shaking (200 rpm, IS-971R, Jeio Tech) at 30°C. Cells were collected in mid-log phase (OD_{600} = 0.5) and the whole culture was spun down (2,000 g, 4°C, 15 min). Cells were washed 2x in ice-cold 100 mM potassium phosphate buffer of pH 7.1 and the pellets were lysed with 200 uL of B-PER Bacterial Protein Extraction Reagent, 0.2 µL of DNAse I (2.6 U μL^{-1}), and 0.2 µL lysozyme (50 mg mL^{-1}) (all Thermo Scientific) for 15 min at RT with slow agitation. Cell lysates were centrifuged at 21,000 g for 30 min at 4°C and supernatants, termed here as cell-free extracts (CFE), were used for activity determination or stored at -80°C for repeated measurements. Total protein concentration in CFE was measured using the method of Bradford[1] with a commercial kit (Sigma-Aldrich). Crystalline bovine serum albumin (Sigma-Aldrich) was used as a protein standard. All accessory enzymes and the majority of chemicals used in the assays described below were purchased from Sigma-Aldrich.

All enzymatic activities were measured in 96-well microplate format. Activities of xylose isomerase (XylA) and xylulokinase (XylB) were measured as described by Dvořák and de Lorenzo[2]. In the XylA assay, activity is coupled to the consumption of NADH by sorbitol dehydrogenase. The assay mixture contained (final concentrations are denoted in all following assays): 1 mM triethanolamine, 0.5 mM NADH, 0.5 U of sorbitol dehydrogenase, 10 mM $MgSO_4$, and 50 mM D-xylose, and 2.5 µL of CFE, all supplemented with 50 mM Tris-HCl buffer (pH 7.5) to a final volume of 200 µL. The reaction mixture was heated to 30°C, and the reaction started with the addition of NADH. In the XylB assay, activity is coupled to pyruvate kinase and lactate dehydrogenase leading to the consumption of NADH. The reaction mixture contained: 0.5 mM NADH, 2 mM ATP, 2 mM $MgCl_2$, 0.2 mM phosphoenolpyruvate, 10 U of pyruvate kinase, 10 U of lactate dehydrogenase, 10 mM D-xylulose, and 2 µL of CFE, adjusted with 50 mM Tris-HCl buffer (pH 7.5) to a final volume of 200 µL. In this and all following enzyme assays, the mixture was heated to 30°C and the reaction started with the addition of NADH or $NADP^+$.

Activities of 6-phosphogluconate dehydratase (Edd) and 2-keto-3-deoxy-6-phosphogluconate aldolase (Eda) were measured in a combined assay based on the protocol of Stephenson *et al.*[3]. The assay measures pyruvate production from 6-phosphogluconate using lactate dehydrogenase and NADH. The reaction mixture contained: 5 mM dithiothreitol, 0.25 mM $MnCl_2$, 1 U of L-lactate dehydrogenase, 0.5 mM NADH, 3.1 mM 6-phosphogluconate, and 6 µL of CFE, adjusted with 100 mM triethanolamine·HCl buffer (pH 7.6) to a final volume of 200 µL.

The activity of glucose 6-phosphate isomerase (Pgi) was measured following the protocol of Sánchez-Pascuala *et al.* with some modifications[4]. The reaction mixture contained: 66 µl of 100 mM glycylglycine buffer (pH = 7.5), 4 mM D-fructose 6-phosphate, 0.5 mM $NADP^+$, 4 mM $MgCl_2$, 1.5 U of glucose-6-phosphate dehydrogenase, and 1 µL of CFE, adjusted with water to a final volume of 200 µL.

The activity of triose phosphate isomerase (Tpi) was measured following the protocol of Sánchez-Pascuala *et al.* with some modifications[4]. The reaction mixture contained: 1 U of glycerol-3-phosphate dehydrogenase, 2.5 mM D,L-glyceraldehyde 3-phosphate, 0.5 mM

NADH, and 2 µl of 20x diluted CFE, adjusted with 100 mM triethanolamine buffer (pH = 7.6) to a final volume of 200 µL.

The activity of glucose 6-phosphate dehydrogenase (Zwf) was measured following the protocol of Sánchez-Pascuala *et al.* with some modifications[4]. The reaction mixture contained: 1 mM D-glucose 6-phosphate, 5 mM $MgCl_2$, 0.5 mM $NADP^+$, and 2 µL of CFE, adjusted with 100 mM triethanolamine buffer (pH 7.6) to a final volume of 200 µL.

The activity of transaldolase (Tal) was measured based on the protocol described by Zhu *et al.* with minor modifications[5]. The reaction mixture contained: 7.5 mM fructose 6-phosphate, 0.75 mM erythrose 4-phosphate, 1 U of glycerol-3-phosphate dehydrogenase, 2 U of triose phosphate isomerase, 0.5 mM NADH, and 2 µL of CFE, adjusted with 100 mM glycylglycine buffer (pH 7.5) to a final volume of 200 µL.

The activity of transketolase (Tkt) was measured based on the protocol of Sobota and Imlay[6]. The reaction mixture contained: 0.3 mM thiamine pyrophosphate, 1 U of glycerol-3-phosphate dehydrogenase, 10 U triose phosphate isomerase, 1 mM xylulose 5-phosphate, 1 mM ribose 5-phosphate, 0.5 mM NADH, and 5 µL of CFE, adjusted with 100 mM glycylglycine buffer (pH 7.5) to a final volume of 200 µL.

The activity of 6-phosphogluconate dehydrogenase (Gnd) was measured following the protocol of Sánchez-Pascuala *et al.* with some modifications[4]. The reaction mixture contained: 2 mM D-gluconate 6-phosphate, 1 mM $NADP^+$, and 2.5 µL CFE, adjusted with 100 mM glycylglycine buffer (pH 7.5) to a final volume of 200 µL.

The oxidation of NADH (decrease in A_{340}) or the reduction of $NADP^+$ (increase in A_{340}) was measured spectrophotometrically at 340 nm with Infinite M Plex plate reader (Tecan). A molar extinction coefficient of 6.22 mM^{-1} cm^{-1}, representing the difference between the extinction coefficients of NAD(P)H and $NAD(P)^+$, was used for activity calculations. 1 unit (U) of activity corresponds to 1 µmol of a substrate (NADH, NADPH) converted by 1 mg of enzyme per/in 1 min.

Supplementary Method 2. Biofilm formation assay with crystal violet
A standard microtiter plate biofilm assay with crystal violet dye was used to assess biofilm formation capacity of the three compared strains. Strains PD584 (control), PD584 L3, and PD689 tt L1 were grown overnight (5 mL LB medium, 350 rpm, 16 h, 30 °C) and inoculated into 200 uL M9 medium with 2 g L^{-1} xylose and Km to the starting OD_{600} of 0.025 in wells of 96 microtiter plate. Cells were grown for 48 h without agitation at 30°C, then 50 uL of the cell culture from each well was used to measure OD and the rest was gently discarded. The plate was washed with H_2O, dried and 200 uL of 0.1% (w/v) crystal violet (CV) solution in H_2O was added. Plate was incubated at RT for 30 min, CV was discarded, plate washed carefully with H_2O and dried at RT. 200 uL of 33% (v/v) acetic acid was added per well to solubilize remaining CV, absorbance was measured at 595 nm and normalized to OD.

Supplementary Method 3. General cloning procedures
Plasmid DNA was routinely isolated using GeneJET Plasmid Miniprep Kit (Thermo Fisher Scientific), or E.Z.N.A. Plasmid DNA Mini Kit I (Omega Bio-Tek). Genomic DNA was isolated using RTP Bacteria DNA Mini Kit (INVITEK Molecular). The genes of interest were amplified by polymerase chain reaction (PCR) using Q5 high fidelity DNA polymerase (New England BioLabs) according to the manufacturer's protocol. The reaction mixture (50 µL)

further contained polymerase HF buffer or GC buffer in case of a template with a high GC content (New England BioLabs), dNTPs mix (0.2 mM each; Roche), respective primers (0.5 mM each), water, and template DNA. Colony PCR was performed in a 10 µL volume using 2x DreamTaq Green PCR Master Mix (Thermo Scientific) with oligonucleotide primers (0.5 µM each) for strain verification and confirmation of inserts in plasmids. All PCR reactions were carried out in Labcycler Gradient (SensoQuest). All used restriction enzymes were from New England BioLabs. Digested DNA fragments were ligated in a 10 µL reaction using T4 DNA ligase (New England BioLabs) at 16 °C overnight or at RT for 15 min according to the manufacturer´s instructions. PCR products and digested plasmids separated by DNA electrophoresis with 0.8 % (w/v) agarose gels were compared to Quick-Load 1 kb DNA Ladder (New England BioLabs) and visualized using G:Box XT4 Digital Imaging System (Syngene). DNA was purified from PCR mixtures or agarose gels using NucleoSpin Gel and PCR Clean-up (Macherey-Nagel). The purity and concentration of DNA were determined by NanoDrop 2000 (Thermo Fisher Scientific). If needed, DNA was concentrated using DNA 120 SpeedVac Concentrator (Thermo Fisher Scientific). Chemocompetent *E. coli* CC118, DH5α, or CC118λpir cells were transformed with ligation mixtures or plasmid constructs, and individual clones selected on LB agar plates with an antibiotic were used for the preparation of cryogenic glycerol (20% v/v in LB medium) stocks. Plasmid constructs were sequenced by Eurofins Genomics or SEQme Czech Republic. Plasmids were inserted into *P. putida* EM42 by electroporation (voltage of 2.5 kV, capacitance of 25 µF, resistance of 200 Ω) in a 2 mm gap cuvette (Thermo Fisher Scientific) using GenePulser XcellTM (Bio-Rad). To prepare electrocompetent cells, 10 mL of overnight culture was washed at least three times with 0.3 M sucrose solution at room temperature. Lastly, the pellet was resuspended in 200 µL of 0.3 M sucrose and 100 µL of this suspension was used for one electroporation reaction[7]. After the electric pulse, 0.9 mL of LB medium was added to the cell suspension (100 µL) in the cuvette and the mixture was transferred to a 2 mL plastic tube. Cells were incubated for 2 h in case of electroporating pSEVA2213, pEMG, pSNW2 and pSW-I plasmids or 5 h in case of pBAMD constructs (200 RPM, 30 °C, NB205 incubator). Alternatively, plasmid constructs were transferred from *E. coli* donors to *P. putida* EM42 by triparental mating, using *E. coli* HB101 helper strain with pRK600 plasmid (Supplementary Table 3). After electroporation or mating, the cells were plated on selective LB agar plates with an antibiotic, incubated at 30 °C overnight and single colonies of transformants or transconjugants were then re-streaked twice on fresh LB plates with antibiotic and the presence of a plasmid was verified by colony PCR and restriction analysis.

Supplementary Method 4. Cloning of transaldolase tal gene (PP_2168) from *P. putida* EM42

The *tal* gene was cloned from the isolated genomic DNA of *P. putida* EM42 into plasmid pSEVA438 with the XylS/Pm expression system inducible with 3-methylbenzoate using the USER cloning[8]. The *tal* gene and pSEVA438 plasmid backbone were amplified using Phusion U Hot Start DNA Polymerase (Thermo Fisher Scientific) with tal Pp U fw/rv and pS438 U fw/rv primer pairs, respectively (Supplementary Data 8), following the polymerase manufacturer´s protocol. The *tal* gene was cloned with a 45 bp upstream sequence containing its native Shine-Dalgarno sequence (Supplementary Data 9). Amplification primers were designed using AMUSER 1.0 software (https://services.healthtech.dtu.dk/services/AMUSER-1.0/), the annealing temperature T_a during PCR was 71°C for the pS438 U fw/rv primer pair and 56°C for the tal Pp U fw/rv primer pair. The USER Enzyme and other enzymes used in the USER cloning protocol (T4 polynucleotide kinase, T4 ligase, *Dpn*I) were from New England Biolabs. PCR products were cleaved with *Dpn*I to remove methylated DNA and 1,000 ng of insert and backbone vector were mixed in T4 ligation buffer with 1U of USER Enzyme. This

mixture was incubated for 30 min at 37 °C then for 30 min at 25 °C and finally 5 µl was transformed into competent *E. coli* CC118.

Supplementary Method 5. Introduction of Ser552Pro mutation into rpoD gene of strain PD580 and PD689

The TCG codon encoding Ser552 was changed for CCG codon encoding Pro in the *rpoD* gene of strains PD580 and PD689 using plasmids pSNW2 and pQURE1 (Supplementary Data 6) and modified homologous recombination-based protocol[9]. Plasmid pSNW2 bearing the mutation was used to cointegrate into the genome. Plasmid pQURE1 was used to produce double-strand breaks and thereby induce homologous recombination-based repair with a 1:1 chance of receiving a WT or mutated sequence.The homologous regions upstream and downstream of the to-be-introduced point mutation T→C (1654 bp) in *rpoD* gene (PP_0387) in *P. putida* KT2440 were amplified using the rpoDmutbase and rpoDHR primer pairs (Supplementary Data 8). The mutated base was included in the rpoDHR primers. The two fragments were assembled together with linearized pSNW2 vector using in-vivo cloning[10]. The rpoD_ingenome primers (Supplementary Data 8) were used to check for cointegrates and ensuing mutated colonies in combination with restriction analysis by *XmaI* restriction enzyme (New England Biolabs) since the mutation resulted in the emergence of a restriction site. Q5 polymerase was used for all amplification intended for vector construction. For analysis, DreamTaq 2x Master Mix was used. For all amplifications, GC enhancer from the Q5 polymerase kit was added to the reactions.

Supplementary Method 6. Analysis of phenazines

For phenazine production assays, pJNN_*phzA1-G1*, *phzM*, *phzS* plasmid with genes encoding a pyocyanin production pathway[11] was transferred into selected xylose-consuming *P. putida* EM42 strains by electroporation. Production of pyocyanin was assayed as described in Bator *et al*. with modifications[11]. Cultures were grown in 2.5 mL of LB medium with gentamycin (10 µg mL^{-1}) and kanamycin (50 µg mL^{-1}) overnight at 30 °C with shaking (220 rpm, NB-205, N-BIOTEK). After centrifugation (2,500 g, RT, 10 min), the pellet was washed in M9 medium and fresh M9 medium (10 mL in 50 mL Erlenmeyer flask) with 0.1 mM salicylic acid, 2 g L^{-1} xylose, gentamycin (10 µg mL^{-1}) and kanamycin (50 µg mL^{-1}) was inoculated to starting OD_{600} of 0.1. Bacteria were cultivated at 30 °C with shaking (200 rpm, JEIO TECH IS-971R, Moris Technology). After 24 h, samples of the culture (1 mL) were withdrawn and measurements were performed. Samples were centrifuged (2,000 g, RT, 10 min) and the absorbance of the supernatant was measured at 691 nm using UV/VIS spectrophotometer Genesys 180 (Thermo Scientific). The concentration of pyocyanin was calculated using Lambert–Beer's law with a molar extinction coefficient of 4.31 mM^{-1} cm^{-1} and absorbance at 691 nm[12].

Supplementary Method 7. Calculations of dry cell weight and growth parameters

For biomass yield ($Y_{X/S}$) calculations, biomass was determined as dry cell weight (DCW). Based on the previously prepared standard curve, one OD_{600} unit determined in *P. putida* EM42 culture in M9 medium is equivalent to 0.38 g L^{-1} of DCW[13]. Important culture parameters were determined as described by Long and Antoniewicz[14]. Specific growth rate (µ) in shake flask experiments was determined during exponential growth by plotting the natural logarithm of biomass concentration versus time and quantifying the slope from regression analysis. Biomass yield ($Y_{X/S}$, where X represents biomass and S represents substrate) was calculated by plotting biomass concentration versus substrate concentration and quantifying the slope from regression analysis. Biomass-specific substrate uptake rate (q_s) was determined during exponential growth using equation **1**:

$$q_s \text{ (mmol } g_{DCW}^{-1} h^{-1}) = \mu \text{ } (h^{-1}) \text{ / } Y_{X/S} \text{ } (g_{DCW} \text{ mmol}^{-1}) \quad \textbf{(1)}$$

Maximal specific growth rate (µmax) and lag phase (in h) were calculated for cultures grown in 48-well microplates using the default settings of The deODorizer program[15].

Supplementary Method 8. Analysis of polar metabolites by capillary ion chromatography-mass spectrometry (IC-MS)

Chemicals and Materials: Acetonitrile in LC-MS grade was obtained from VWR International GmbH. All other reagents were obtained from Sigma-Aldrich. Purified water from a Milli-Q-Academic system was used throughout the sample preparation and the measurements. The standards utilized for method development are listed in Supplementary Table 4. The samples were reconstituted in purified water immediately before analysis.

Capillary IC-MS analysis: Capillary IC-MS analysis was performed using a Dionex ICS-4000 Capillary HPIC system connected to a Q Exactive Plus (Thermo Fisher Scientific) utilizing a HESI-II electrospray ionization (ESI) source. Sample injections were performed by a Dionex AS-AP autosampler. For delivery of a regeneration water flow and a make-up solution flow, the system was equipped with two external AXP Auxiliary pumps from Thermo Fisher Scientific.

Samples were separated on a Dionex IonPac AS11-HC-4µM column (250 x 0.4 mm, 4 µm; Thermo Fisher Scientific) that was maintained at 35°C. The flow rate was set to 17 µL min^{-1} and the injection volume was 0.4 µL. The KOH-gradient program utilized for the separation was: 1 mmol L^{-1} KOH held for 2 min, increased to 15 mmol L^{-1} at 8 min, 20 mmol L^{-1} at 12 min, 30 mmol L^{-1} at 22 min, 70 mmol L^{-1} at 37 min held for 3 min and finally increased to 100 mmol L^{-1} at 41 min held for 2 min followed by a 2 min decrease back to initial conditions, which was held for 5 min. The total analysis time was 50 min. For an improved ionization, an acetonitrile/water solution (1:1) containing 0.1 Vol.-% ammonium hydroxide was delivered as make-up flow at a flow rate of 30 µL min^{-1} and combined with the eluent via a low dead volume mixing tee, and passed through a grounding union before entering the ESI source.

The Q Exactive mass spectrometer was operated in negative ionization mode and the spray voltage was set to 2.8 kV. The capillary temperature was set to 250 °C, the sheath gas flow rate was 25 (arbitrary units), the auxiliary gas flow rate was 8 (arbitrary units), the sweep gas flow rate was 0 (arbitrary units), and the S-lens level was set to 50.

The samples were measured in targeted selected ion monitoring (tSIM) mode with the following parameters for the analysis of the cell extracts: resolution, 140,000 (at *m/z* 200); auto gain control target, 1×10^5; maximum ion injection time, 100 ms; and an isolation window with the mass range of 7 *m/z* to include the ^{13}C-labelled isotopes. The exact masses for the unlabelled metabolites and the measuring time for each tSIM-window were experimentally evaluated with authentic standards. To include isotopes with multiple incorporated ^{13}C-atoms and to centre the tSIMs' mass range on the ^{13}C-labelled isotopes, the measured accurate masses from the authentic standards were expanded by 3 *m/z* for the inclusion list. The targeted masses and retention time windows are listed in Supplementary Table 5.

For IC-MS instrument control, Xcalibur 4.1 software and the SII plugin (Thermo Scientific) was utilized. Metabolites were assigned by accurate mass (< 10 ppm relative mass deviation) and retention times by comparison to authentic standards. Identification and quantification of metabolites and the ^{13}C-labelled isotopes was performed on the basis of extracted ion chromatograms (relative mass deviation of ±10 ppm) of each isotope by peak area determination with Xcalibur QualBrowser software.

Supplementary Method 9. Whole-genome sequencing

P. putida EM42-derived strains were cultured in LB medium at 30°C till mid-exponential phase. Cells were collected and enzymatically treated as previously described[16] with the following steps and modifications: First, 10 mL of bacterial culture in mid-exponential phase cultivated in LB at 30°C was centrifuged at 3,000 × g and 10°C for 10 min, washed with 5 mL of wash solution (10 mM Tris-HCl, 10 mM EDTA, 10 mM EGTA, 1 M NaCl of pH 7.5), and resuspended in Tris-EDTA (TE) buffer with achromopeptidase (1,000 U mL^{-1}; Sigma-Aldrich), lysozyme (5 mg mL^{-1}; Sigma-Aldrich), and RNase A (200 µg mL^{-1}; New England BioLabs) in a total volume of 500 µL, followed by incubation for 1 to 2 h at 37°C until lysis appeared. Then, 30 µL of 10% SDS and 5 µL of proteinase K (20 mg mL^{-1}; Sigma-Aldrich) were added, and the sample was incubated for 60 min at 50°C. Genomic DNA was extracted from cell lysate using the Genomic DNA Clean & Concentrator-25 kit (Zymo Research) according to the manufacturer's instructions. For Oxford Nanopore sequencing, the library was prepared using the SQK-RAD004 Rapid Sequencing kit (Oxford Nanopore Technologies) according to the manufacturer's instructions. The library was sequenced with a FLO-FLG001 flow cell (R9.4.1) in a MinION device controlled by MinKNOW software v.23.07.2, (Oxford Nanopore Technologies), which was also used for basecalling (super-accurate model with minimum q-score threshold of 10), demultiplexing, and barcode trimming. For Illumina-based sequencing, a 500-bp sequencing library was prepared with xGen™ DNA Lib Prep EZ (Integrated DNA Technologies) according to the manufacturer's instructions. In brief, 500 ng total DNA was enzymatically fragmented for 8 minutes. CS_UMI Adapter (Integrated DNA Technologies) and dual indexing primers were used to adapt the library. The library was amplified for 8 PCR cycles. After QC and pooling, the library was sequenced using Illumina Nextseq instrument with 300 cycles mid output chemistry. Illumina reads were trimmed and filtered using Trimmomatic v0.38.1 with the sliding window model using a required average quality of 20[17]. Complete bacterial genome sequences were obtained using a hybrid assembly with Unicycler v0.4.8[18] with minimal k-mer size of 0,2 and highest k-mer size of 0,95 with 10 k-mer steps used in SPAdes assembly. The resulting assembly was polished with Pilon v1.24[19]. Assembly of Nanopore-only reads was performed using Flye v.2.9.1[20] and Medaka consensus pipeline v1.7.2 (Oxford Nanopore). Prokka v1.14.6 was used to annotate gene products for in-house proteomic analysis[21]. The sequence of whole plasmid pSEVA2213_*xylABE* isolated from individual *P. putida* EM42 mutants was verified separately by Plasmidsaurus service (USA) using Oxford Nanopore technology (https://www.plasmidsaurus.com). Genomic data were handled by Geneious Prime 2022.2.2 (Biomatters). Mapping of sequencing reads was performed using Minimap2 (for Nanopore reads) and Bowtie (for Illumina reads) Plugins, and whole-genome alignment was performed using Mauve Plugin. Variant calling using Illumina reads was performed as follows: reads were mapped to reference sequences of strain PD584 and its plasmid pPD584 using Bowtie2 ("End to end" alignment type and "Medium sensitivity" preset), Minimum Variant Frequency = 0.1, Maximum Variant *p*-value = 1E-12, Minimum Strand-Bias *p*-value = 1E-2 when exceeding 65% bias. Variant calling using Nanopore reads was performed as follows: reads were mapped to the PD689 reference genome using Minimap2 (K-mer length = 28), Minimum Variant Frequency = 0.1, Maximum Variant *p*-value = 1E-12, Minimum Strand-Bias *p*-value = 1E-2 when exceeding 65% bias, and Homopolymer Quality Reduction = 30% because of problematic detection of homopolymers by the Nanopore platform. Only polymorphisms affecting protein sequences were considered in the case of the chromosomal sequences, while in plasmid, polymorphisms in the whole sequence were considered.

All sequencing data and assembled whole-genome sequences were deposited under NCBI BioProject PRJNA914626. The whole-genome sequences and raw sequencing data have been deposited in the GenBank database and SRA database (NCBI), respectively, under accession numbers summarized in Supplementary Table 1. The genomes were annotated using the NCBI Prokaryotic Genome Annotation Pipeline[22].

Supplementary Method 10. Proteomic analyses

P. putida EM42 strains were pre-cultured overnight in 2.5 mL of LB medium with kanamycin. Cells were spun down (2,000 rpm, RT, 7 min), washed with M9 medium, and used for the inoculation of main cultures in 250 mL shake flasks with 50 mL of M9 medium, 2 g L^{-1} xylose or glucose and kanamycin to a starting OD_{600} of 0.05 (200 rpm, IS-971R, Jeio Tech). Cells (25 mL) were collected in the mid-exponential phase (OD_{600}=0.5) in pre-chilled falcon tubes (2,500 g, 4°C, 5 min), washed twice with ice-cold PBS buffer (per 1 L: 8 g NaCl, 0.2 g KCl, 1.44 g Na_2HPO_4, 0.24 g KH_2PO_4, pH 7.4) and pelleted in 1.5 mL Eppendorf tubes (15,000 g, 4°C, 2 min). Cell pellets were immediately frozen at -80°C and kept frozen until further use.

Sample preparation and LC-MS analyses: Cell pellets were lysed using SDT buffer (4 % sodium dodecyl sulfate, 0.1 M dithiotreitol, 0.1 M Tris/HCl, pH 7.6) at 95°C for 30 min and the resulting protein solution was cleared by centrifugation at 20,000 g for 15 min. The protein lysates were processed by filter-aided sample preparation (FASP)[23] with some modifications as specified. The samples were mixed with 8 M UA buffer (8 M urea in 100 mM Tris-HCl, pH 8.5), loaded onto the Microcon device with MWCO 30 kDa (Merck Millipore), and centrifuged (7,000 g, 20°C, 30 min). The retained proteins were washed (all centrifugation steps after sample loading were performed at 14,000 g) with 200 µL UA buffer. The washed protein concentrates kept in the Microcon device were mixed with 100 µL of UA buffer containing 50 mM iodoacetamide and incubated in the dark for 20 min. After the next centrifugation step, the samples were washed three times with 100 µL of UA buffer and three times with 100 µL of 50 mM $NaHCO_3$. Trypsin (sequencing grade, Promega) was added onto the filter and the mixture was incubated for 18 h at 37°C (enzyme:protein ratio 1:100). The tryptic peptides were eluted by centrifugation followed by two additional elutions with 50 µL of 50 mM $NaHCO_3$. Peptides were then cleaned by liquid-liquid extraction (3 iterations) using water-saturated ethyl acetate[24]. Cleaned FASP eluate was evaporated completely in SpeedVac concentrator (Thermo Fisher Scientific). The resulting peptides were extracted into LC-MS vials by 2.5% formic acid (FA) in 50% acetonitrile (ACN) and 100% ACN with the addition of polyethylene glycol (20,000; final concentration 0.001%)[25] and concentrated in a SpeedVac concentrator (Thermo Fisher Scientific). LC-MS/MS analyses of all peptide mixtures were done using RSLCnano system connected to Orbitrap Exploris 480 spectrometer (Thermo Fisher Scientific) with EASY Spray ion source (Thermo Fisher Scientific) installed. Prior to LC separation, tryptic digests were online concentrated and desalted using a trapping column (300 µm × 5 mm, µPrecolumn, 5 µm particles, Acclaim PepMap100 C18, Thermo Fisher Scientific). After washing the trapping column with 0.1% FA, the peptides were eluted (flow 300 nL min^{-1}) from the trapping column onto Acclaim PepMap RSLC C18 column (2 µm particles, 75 µm × 250 mm; Thermo Fisher Scientific) by 104 min long gradient. Mobile phase A (0.1% FA in water) and mobile phase B (0.1% FA in 80% acetonitrile) were used in both cases. The gradient elution started at 3% of mobile phase B and increased from 3% to 37% during the first 94 min, then increased linearly to 80% of mobile phase B in the next 7 min and remained at this state for the next 3 min. Equilibration of the trapping column and the column was done prior to sample injection into the sample loop. The analytical column outlet was directly connected to the Easy Spray ion source.

Data was acquired in a data-independent acquisition mode (DIA). The survey scan covered the *m/z* range of 350-1,400 at a resolution of 60,000 (at *m/z* 200) and a maximum injection time of 55 ms. HCD MS/MS (27% relative fragmentation energy) was acquired in the range of *m/z* 200-2000 at 30,000 resolution (maximum injection time 55 ms). An overlapping windows scheme in *m/z* range from 400 to 800 was used as the isolation window placements.

Analysis of proteomics data: DIA data were processed in DIA-NN version 1.8[26] in library-free mode against the modified cRAP database (based on http://www.thegpm.org/crap/; 112 sequences in total) and UniProtKB protein database for *P. putida* (number of protein sequences: 310,975) and UniProtKB protein database for *E. coli* (number of protein sequences: 4,402). No optional, carbamidomethylation as fixed modification and trypsin/P enzyme with 1 allowed missed cleavages and peptide length 7-30 were set during the library preparation. False discovery rate (FDR) control was set to 1%. MS1 and MS2 accuracies as well as scan window parameters were set based on the initial test searches (median value from all samples ascertained parameter values). MBR was switched on. Protein intensities reported in the DIA-NN main report file (Supplementary Data 2) were further processed using the software container environment (https://github.com/OmicsWorkflows), version 4.1.3a. The processing workflow is available upon request. Briefly, it covered: a) removal of low-quality precursors and contaminant protein groups, b) protein group intensities log2 transformation, c) LoessF normalization, d) filtering out of protein groups not quantified in more than half of the replicates of at least one sample type (e) imputation of the missing values from the random distribution around the global minimal value, f) normalized and imputed protein intensities were used for differential expression using LIMMA statistical test and g) proteins with adjusted *p*-value <0.05 (*p* values adjustment on multiple hypothesis testing was done using Benjamini & Hochberg method) and log2 fold change >1 were used for Volcano plots.

Duplicities were removed in Microsoft Office Excel. Gene identifiers were coupled to UniProt identifiers using the ID mapping tool available at uniprot.org. Data were visualized in Escher (escher.github.io) using a genome-scale model iJN1463 of *P. putida* KT2440[27].

Targeted proteomics for monitoring of specific peptide forms: Potential forms of a short leading peptide that could be generated in PD584 L3 as a consequence of the identified duplication upstream of the *xylA* gene in pSEVA2213_*xylABE* plasmid (as described in the main body of the manuscript) were searched through targeted proteomics. Samples for the proteomic analysis were prepared as described above with some modifications. After washing the samples of protein lysates loaded onto the Microcon device and centrifuged with UA buffer with 50 mM $NaHCO_3$, flowthrough was loaded onto Vivacon 500 MWCO 2kDa (Sartorius), washed with 100 µL UA buffer and after that with 100 µL of 50 mM $NaHCO_3$. Peptides were eluted from filter by 2x 50 µL of 50 mM $NaHCO_3$, 15 min, laboratory temperature, 750 rpm, centrifuge upside down 1,000 g, 3 min. Eluates were evaporated completely in SpeedVac concentrator (Thermo Fisher Scientific). Resulting peptides were extracted into LC-MS vials by 2.5% formic acid (FA) in 50% acetonitrile (ACN) and 100% ACN with addition of polyethylene glycol (20,000; final concentration 0.001%) and concentrated in a SpeedVac concentrator (Thermo Fisher Scientific). LC-MS/MS analyses of all peptide mixtures were done using RSLCnano system connected to Orbitrap Exploris 480 spectrometer (Thermo Fisher Scientific) with EASY Spray ion source (Thermo Fisher Scientific) installed. Prior to LC separation, tryptic digests were online concentrated and desalted using trapping column (300 µm × 5 mm, µPrecolumn, 5µm particles, PepMap Neo Trap Cartridge, Thermo Fisher Scientific). After washing of trapping column with 0.1% FA, the peptides were eluted (flow 200 nL min^{-1}) from the trapping column onto separation column (Aurora C18, 1.7 µm particles,

75 µm × 250 mm, P/N AUR3-25075C18-TS; IonOpticks) operated at 50°C by 66 min long gradient (0min: 3% B, 45 min: 42% B, 66 min: 80% B; mobile phase A: 0.1% FA in water; mobile phase B: 0.1% FA in 80% acetonitrile). MS data were acquired in a product ion scan mode with additional survey scan measurement. The product ion scan mode was set for the expected peptide forms (Supplementary Table 6) monitored during the whole LC-MS run. Product ion spectra were set as follows: custom AGC target of 3,000 %, automatic maximal injection time mode, isolation width 1.2Th, relative fragmentation energy 30%, orbitrap analyser resolution 60,000, first mass *m/z* 120.

Supplementary Note 1. Growth of selected engineered and evolved strains on glucose

We tested the growth of the three evolved strains PD584 L3, PD584 tt L3, and PD689 tt L1 and their ancestors PD310, PD589 and PD689 on glucose (Supplementary Fig. 7a). Glucose is the most abundant hexose sugar in (hemi)cellulose and its simultaneous utilization with xylose is a desirable property of any microbial cell factory applicable in lignocellulose biorefineries[2,28,29]. The control strains (PD310, PD584, PD689) and PD584 L3 all grew well on glucose ($\mu \geq 0.50$ h^{-1}, growth lag ~ 1.0 h) with PD584 and PD584 L3 being the fastest (0.58 ± 0.01 h^{-1} and 0.57 ± 0.00 h^{-1}, respectively). On the other hand, two evolved strains PD689 tt L1 and PD584 tt L3 showed reduced growth rate (0.42 ± 0.01 h^{-1} and 0.45 ± 0.01 h^{-1}, respectively) and a prolonged lag phase (2.21 ± 0.20 h and 4.47 ± 0.15 h, respectively). Therefore, it seems that in the case of the slower-growing strains, tailoring of the PPP and subsequent ALE on xylose affected the metabolism of glucose. Further growth experiments with PD584 L3 and PD689 tt L1 confirmed that both new strains also maintained the previously reported ability of the ancestral PD310 to co-utilize glucose and xylose (Supplementary Fig. 7b)[2].

Supplementary Note 2. Whole-genome sequencing of engineered and evolved strains

Firstly, the selected engineered and evolved strains PD584 L3, PD584 tt L3, and PD689 tt L1 together with the reference strains PD584 and PD689 and a slower-growing mutant PD584 ttrr L2 were sequenced using Oxford Nanopore technology (Supplementary Method 9, Supplementary Table 1). We aimed to verify the chromosomal integration of the expression cassettes *talB-tktA* and *talB-tktA-rpe-rpiA*. The sequencing identified the locus of the cassette integration in PD689 tt L1 (PP_1181, which encodes a two-component system response regulator from OmpR family) and in PD584 ttrr L2 (PP_1145, which encodes RNA polymerase-associated protein RapA) and confirmed the absence of the cassette in controls PD584 and PD689 and in the evolved strain PD584 L3. The unstable phenotype of PD584 ttrr L2 strain (reflected by the fact that this clone was not able to maintain the desired phenotype across different cultivation formats, Supplementary Fig. 4) can be related to the integration of the *talB-tktA-rpe-rpiA* cassette in the *rapA* locus. RapA is a transcription regulator that stimulates RNA polymerase recycling and the disruption of the corresponding gene may thus cause divergence in global gene expression pattern[30]. Surprisingly, sequencing revealed that the *talB-tktA* cassette was absent in the genome of the PD584 tt L3 strain. The cassette may have been lost during ALE or its integration into the chromosome by the Tn5 minitransposon system may have failed altogether.

The genomes of the reference strain PD584 and the two best xylose-utilizing strains from ALE, PD584 L3 and PD689 tt L1, were further sequenced by Illumina platform and complete whole-genome sequences were determined using hybrid assembly (Supplementary Method 9, Supplementary Table 1). For the reference strain PD689, the whole-genome sequence was determined using Oxford Nanopore sequencing data only. Subsequently, we verified the sequence of the *talB-tktA* expression cassette integrated into the chromosome of strain PD689 tt L1. Four silent mutations (three in *talB* and one in *tktA*) and one missense mutation (GCA→ACA resulting in Ala247→Thr247 substitution in TalB) were identified. The Ala247→Thr247 substitution is relatively distant from the binding pocket and can be found in functional TalB variants, for instance in the PDB crystal structure 4S2C[31]. Hence, we do not expect it to have a negative effect on the enzyme's activity.

Supplementary Table 1. Accessions to sequencing data of *Pseudomonas strains* in this work in NCBI databases.

BioProject PRJNA914626			
Strain	BioSample accession	GenBank accession	SRA accession[1]
PD584	SAMN32340832	CP115665 - chromosome<br>CP115666 - plasmid	SRR23032142<br>SRR23032143<br>SRR23032144
PD584 L3	SAMN32340833	CP115663 - chromosome<br>CP115664 - plasmid	SRR23032140<br>SRR23032141
PD689	SAMN38086568	CP137886 - chromosome	SRR26690099
PD689 tt L1	SAMN32340834	CP115661 - chromosome<br>CP115662 - plasmid	SRR23032137<br>SRR23032138<br>SRR23032139
EM42 Δ*gcd*		-	SRR22840563
EM42 Δ*gcd* Col. 2A		-	SRR26662873
EM42 Δ*gcd* Col. 2B	SAMN32340957	-	SRR26662872
EM42 Δ*gcd* Col. 2C		-	SRR26662871
EM42 Δ*gcd* Col. 2D		-	SRR26662870
PD310	SAMN32340958	-	SRR22840562
PD584 tt L3	SAMN32340959	-	SRR22840561
PD584 ttrr L2	SAMN32340960	-	SRR22840560
PD580	SAMN38324436	-	SRR26883051

[1] SAR concern the whole genome, i.e. chromosomal and plasmid sequences.

Supplementary Table 2. Presence of multiplied genomic locus PP_2114 - PP_2219 in the chromosome of sequenced *Pseudomonas putida* strains.

	Coverage										
	Chromosome without multiplied region (A)				Multiplied region (B)				B/A ratio		
**Strain**	**Illumina**	**SD**	**Nanopore**	**SD**	**Illumina**	**SD**	**Nanopore**	**SD**	**Illumina**	**Nanopore**	**avg.**
**PD580**	N/A	N/A	12.8	3.9	N/A	N/A	11.9	3.4	N/A	0.9	**0.9**
**PD584**	107.4	23.3	34.2	9.1	768.6	131.8	161.8	12.5	7.2	4.7	**5.9**
**PD584 L3**	56.2	14.2	27.4	8.8	386.7	69.4	148.6	14.5	6.9	5.4	**6.2**
**PD689**	N/A	N/A	2.3	1.5	N/A	N/A	1.8	1.1	N/A	0.8	**0.8**
**PD689 tt L1**	425.9	66.1	30.9	6.6	388.8	47.8	29.3	6.0	0.9	0.9	**0.9**
**PD310**	N/A	N/A	4.6	2.5	N/A	N/A	17.1	5.9	N/A	3.7	**3.7**
**EM42 Δ*gcd***	N/A	N/A	6.4	2.5	N/A	N/A	5.7	2.6	N/A	0.9	**0.9**
**EM42 Δ*gcd* Col. 2A**	N/A	N/A	2.3	1.5	N/A	N/A	13.9	3.8	N/A	6.0	**6.0**
**EM42 Δgcd Col. 2B**	N/A	N/A	10.5	3.6	N/A	N/A	33.7	5.4	N/A	3.2	**3.2**
**EM42 Δ*gcd* Col. 2C[1]**	N/A	N/A	21.9	6.9	N/A	N/A	38.7	7.6	N/A	1.8	**1.8**
**EM42 Δ*gcd* Col. 2D[2]**	N/A	N/A	26.0	6.6	N/A	N/A	55.5	6.7	N/A	2.1	**2.1**

N/A - was not performed. Coverage was determined by Minimap2 plugin for Nanopore platform and Bowtie plugin for Illumina platform (Geneious Prime 2022.2.2). [1]118-kb region bordered by ISP8 elements in pos. 2,372,035 - 2,490,113 bp in the reference genome of PD584; in strain 2C, the 446-kb multiplied region between ISP8 elements was detected (pos. 2,044,264 bp - 2,490,062 bp in the reference genome); and in strain 2D[2], the 275-kb multiplied region between ISP8 elements was detected (pos. 2,214,987 bp - 2,490,164 bp in the reference genome). Both larger multiplied regions include the 118-kb region.

Supplementary Table 3. ***Escherichia coli*** **strains used in this study.**

Strain	Characteristics	Reference
Dh5α	Cloning host: F-λ-*endA1 glnX44*(AS) *thiE1 recA1 relA1 spoT1 gyrA96*(NalR) *rfbC1 deoR nupG* Φ80(*lacZ*Δ*M15*) Δ(*argF-lac*)*U169 hsdR17*($r_K^- m_K^+$)	[32]
CC118	Cloning host: Δ*(ara-leu) araD* Δ*lac X174 galE galK phoA thiE1 rpoB(Rif^R) argE(Am) recA1*	[33]
CC118λ*pir*	Cloning host: *araD139* Δ*(ara-leu)7697* Δ*lacX74 galE galK phoA20 thi-1 rpsE rpoB*(Rif^R) *argE*(Am) *recA1*, λpir lysogen	[34]
HB101	Helper strain for tri-parental mating: F– λ– *hsdS20*(r_B^- m_B^-) *recA13 leuB6*(Am) *araC14* Δ(*gpt-proA*)62 *lacY1 galK2*(OC) *xyl-5 mtl-1 thiE1 rpsL20*(Sm^R) *glnX44*(AS)	[35]

Supplementary Table 4. List of metabolite standards, their abbreviations and purities.

Compound	Abbreviation	Purity
pyruvic acid sodium salt	pyruvate	≥ 98 %
disodium succinate	succinate	≥ 96 %
DL-malic acid	malate	≥ 99 %
sodium fumarate dibasic	fumarate	≥ 99 %
citric acid monohydrate	citrate	≥ 99 %
DL-isocitric acid trisodium salt	isocitrate	≥ 93 %
α-D-glucose 1-phosphate disodium hydrate	G1P	≥ 97 %
D-glucose 6-phosphate sodium salt	G6P	≥ 99 %
fructose-6-phosphate disodium salt hydrate	F6P	≥ 98 %
D-ribose 5-phosphate disodium salt hydrate	R5P	≥ 98 % (TLC)
6-phosphogluconic acid trisodosium salt	6PG	≥ 95 %
D-(-)-3-phosphoglyceric acid disodium salt	G3P	≥ 93 %
D-fructose 1,6-bisphosphate trisodium salt hydrate	FBP	≥ 97 % (TLC)

Besides the pyruvic acid standard, which was purchased from Merck, all metabolite standards were obtained from Sigma Aldrich Chemie.

Supplementary Table 5. List of evaluated metabolite standards including the retention time and measured accurate mass and the hereon based targeted masses and the defined time windows utilized as inclusion list for targeted selected ion monitoring (tSIM) measurements of *P. putida* extracts.

**Metabolite**	t_R /min	**Exact mass** *m/z*	**Accurate mass** *m/z*	**Targeted mass** *m/z*	**tSIM-window** /min
pyruvate	5.72	87.0077	87.0084	90.0084	4.50-12.00
succinate	11.74	117.0182	117.0193	120.0193	11.00-16.00
malate	11.73	133.0131	133.0134	136.0134	11.00-17.00
fumarate	13.79	115.0026	115.0037	118.0037	12.00-18.00
citrate	20.83	191.0186	191.0199	194.0189	20.00-26.00
isocitrate	21.74				
glucose 1-phosphate	10.65	259.0213	259.0224	262.0224	10.00-18.00
glucose 6-phosphate*	13.23				
fructose 6-phosphate*	13.23				
ribose 5-phosphate	14.02	229.0108	229.0120	232.0120	11.00-18.00
6-phosphogluconate	17.75	275.0163	275.0175	278.0175	15.00-20.00
		137.0039*	137.0051*	140.0051	
3-phosphoglyceric acid	19.47	184.9846	184.9857	187.9857	18.00-24.00
fructose 1,6-bisphosphate	29.61	338.9877	338.9888	341.9888	28.50-35.00

*Glucose 6-phosphate and fructose 6-phosphate could not be separated.

Supplementary Table 6. List of peptide forms monitored during the production mode measurement. "M[+16]" represents oxidized methionine form.

Compound	Precursor (*m/z*)	Precursor charge (*z*)
MQAYSSKRNIPCKPILTSSIAFVMK	938.5007	3
MQAYSSKRNIPCKPILTSSIAFVM[+16]K	943.8323	3
M[+16]QAYSSKRNIPCKPILTSSIAFVM[+16]K	949.1639	3
MQAYSSKR	485.7424	2
M[+16]QAYSSKR	493.7398	2
MQAYSSK	407.6918	2
M[+16]QAYSSK	415.6893	2
QAYSSKRNIPCKPILTSSIAFVMK	894.8205	3
QAYSSKRNIPCKPILTSSIAFVM[+16]K	900.1521	3
QAYSSKR	420.2221	2
QAYSSK	342.1716	2

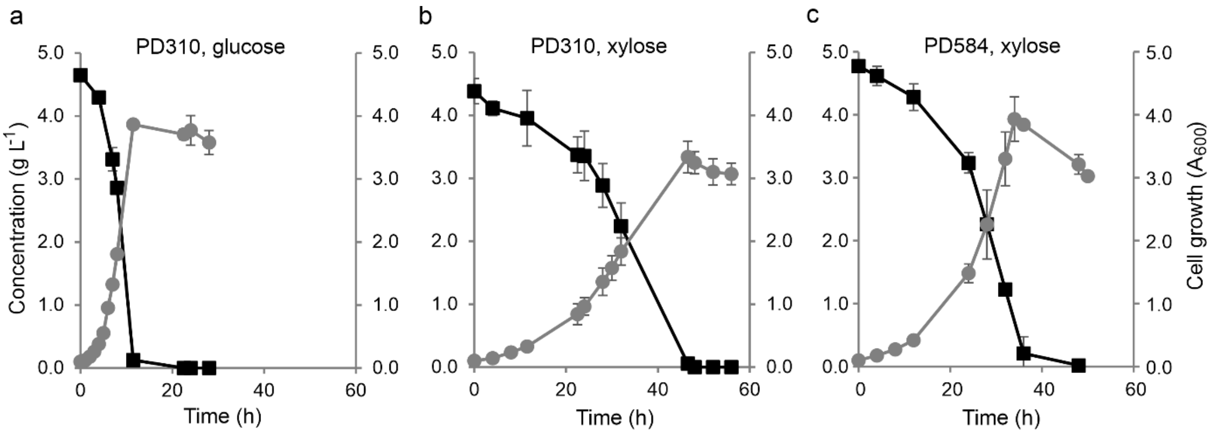

Supplementary Figure 1. Shake flask cultures of engineered *P. putida* strains in M9 minimal medium with 5 g L^{-1} sugar substrate. (**a**) *P. putida* PD310 grown on glucose, (**b**) *P. putida* PD310 grown on xylose, and (**c**) *P. putida* PD584 grown on xylose. Cell growth, grey circles; glucose or xylose, black squares. Data points represent means ± standard deviations calculated from three (n=3) biological replicates. Source data are provided as a Source Data file.

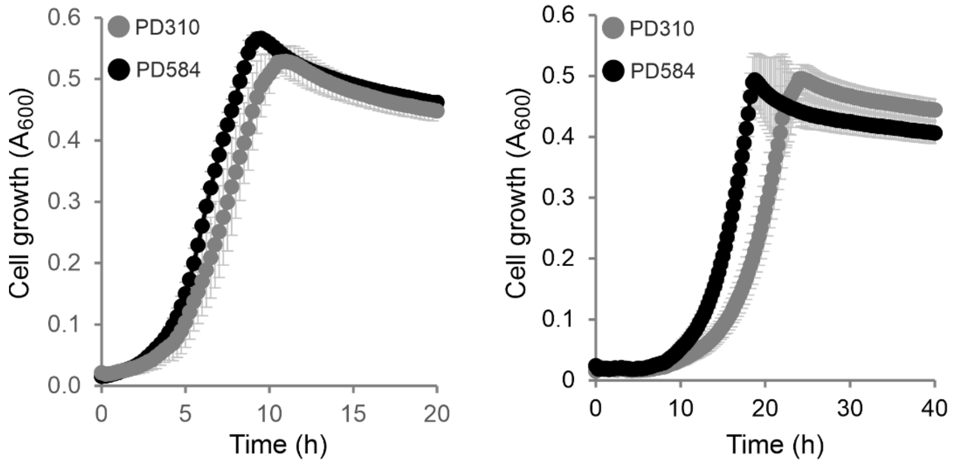

Supplementary Figure 2. Growth of *hexR*$^+$ PD310 and *hexR*$^-$ PD584 in M9 minimal medium with 2 g L^{-1} D-glucose (left graph) or 2 g L^{-1} D-fructose (right graph) in 48-well microplate. Data are shown as a mean ± standard deviation from three (n=3) biological replicates. Source data are provided as a Source Data file.

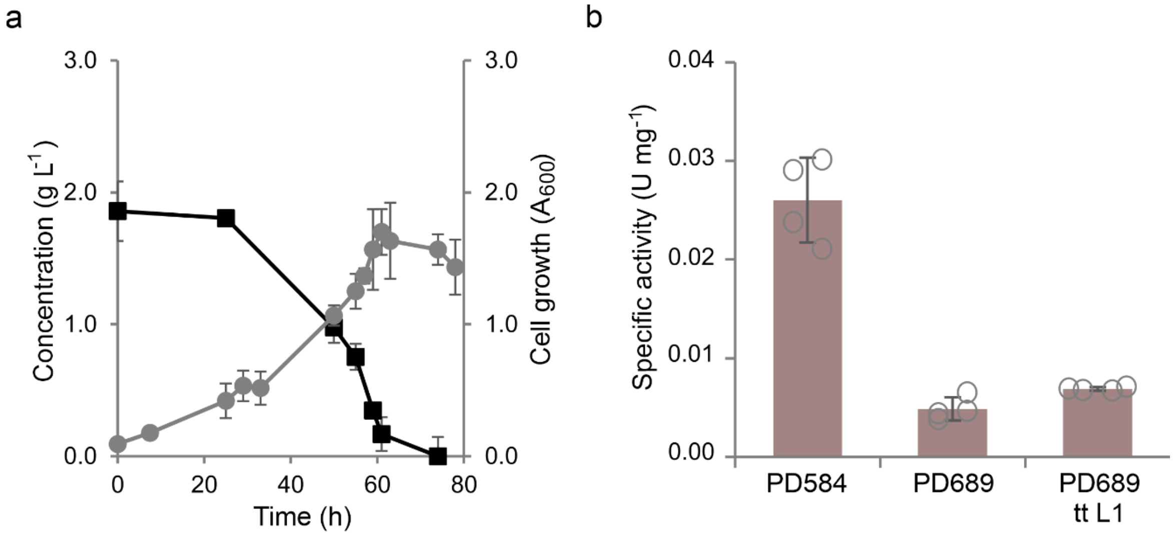

Supplementary Figure 3. Shake flask cultures of *Pseudomonas putida* PD689 (a) in M9 minimal medium with xylose (2 g L^{-1}) and (b) specific activity of 6-phosphogluconate dehydrogenase Gnd determined in cell-free extracts of PD584, PD689, and PD689 tt L1 grown on xylose. (**a**) Cell growth, dark grey circles; xylose, black squares. Data points represent means ± standard deviations calculated from three (n=3) biological replicates. (**b**) Columns represent means ± standard deviations calculated from four (n=4) biological replicates. Source data are provided as a Source Data file.

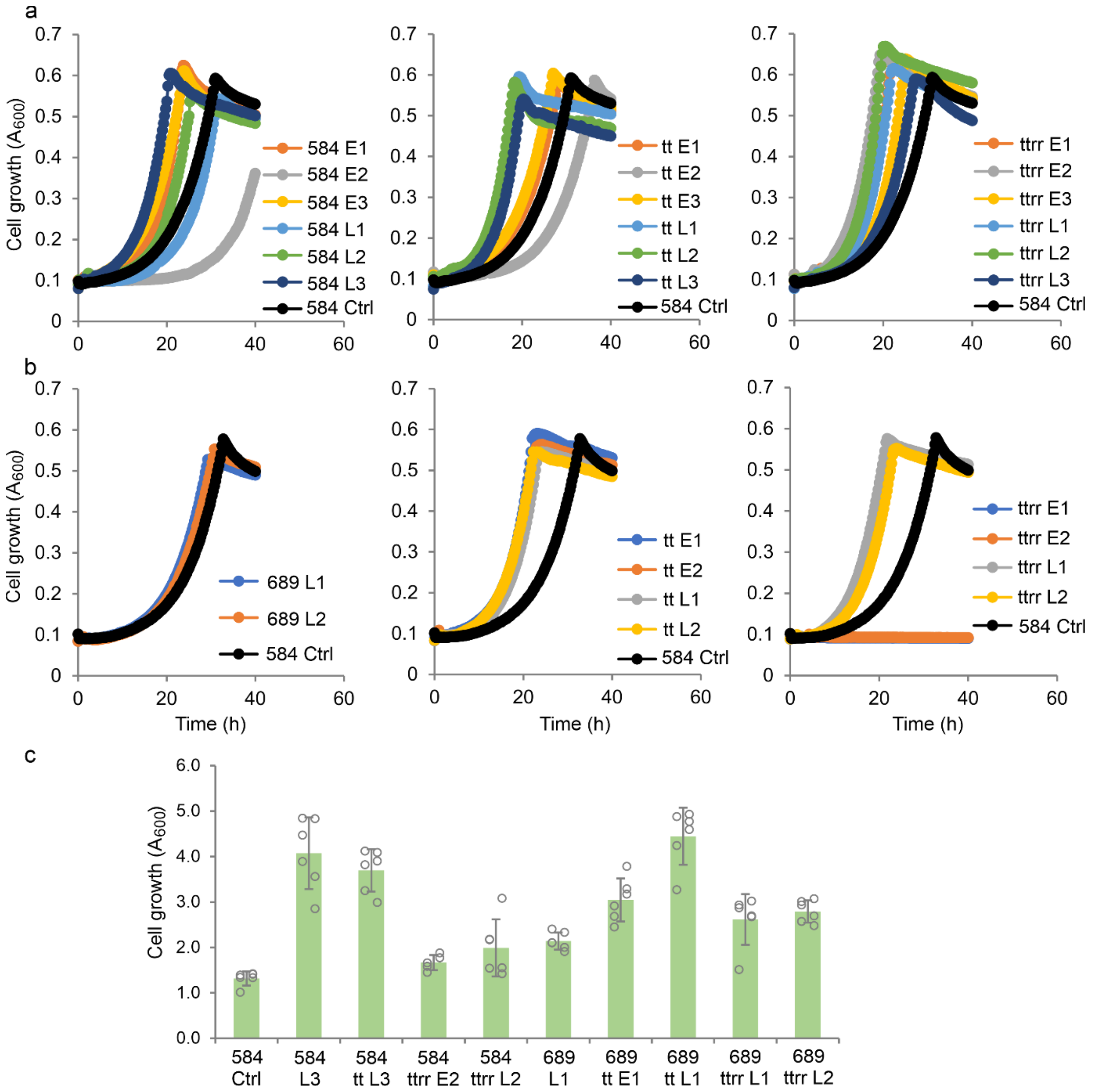

Supplementary Figure 4. Screening of selected evolved mutants of *P. putida* PD584 and PD689 strains expressing heterologous pentose phosphate pathway genes in M9 minimal medium with 2 g L^{-1} xylose in 48-well microplate (a and b) or in shake flasks with 5 g L^{-1} xylose (**c**, A_{600} values determined after 24 h culture). E and L in the figure legends in **a** (PD584-derived candidates) and **a** (PD689-derived candidates) represent clones picked in the earlier phase of the ALE experiment when OD_{600} of a given culture 24 h after inoculation reached the value of at least 3.5 for the first time (E), or clones picked at the end of the ALE experiment (L). Data in (**c**) are shown as mean ± standard deviation from six (n=6) biological replicates from two independent experiments (each of three biological replicates). Source data are provided as a Source Data file.

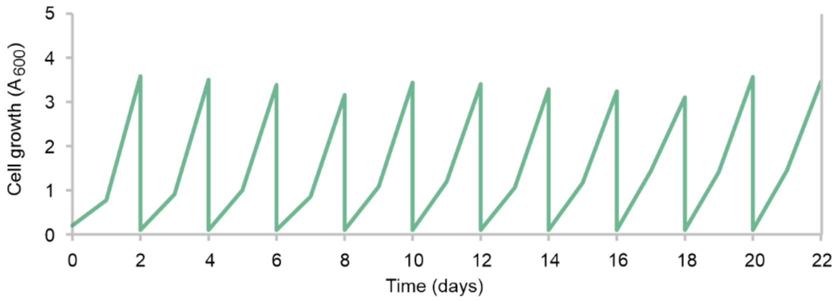

Supplementary Figure 5. Adaptive laboratory evolution (ALE) on xylose of *P. putida* PD310. *P. putida* PD310 was cultured in shake flasks containing 20 mL of M9 minimal salts medium, 5 g L^{-1} D-xylose and kanamycin and passaged every 48 h. Data points represent means of two (n=2) biological replicates. Source data are provided as a Source Data file.

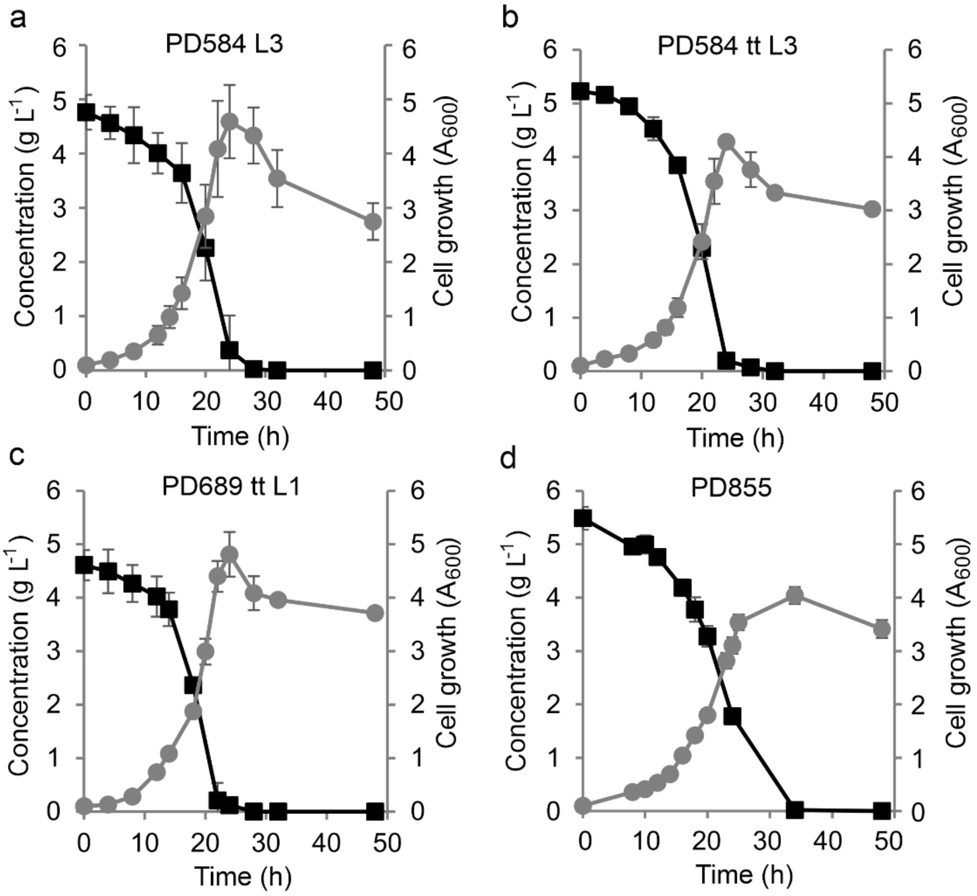

Supplementary Figure 6. **Shake flask cultures of *P. putida* PD584 L3 (a), PD584 tt L3 (b), PD689 tt L1 (c), and PD855 (d) in M9 minimal medium with xylose (5 g L^{-1}).** Cell growth, dark grey circles; xylose, black squares. Data points represent means ± standard deviations calculated from three (n=3) biological replicates. Source data are provided as a Source Data file.

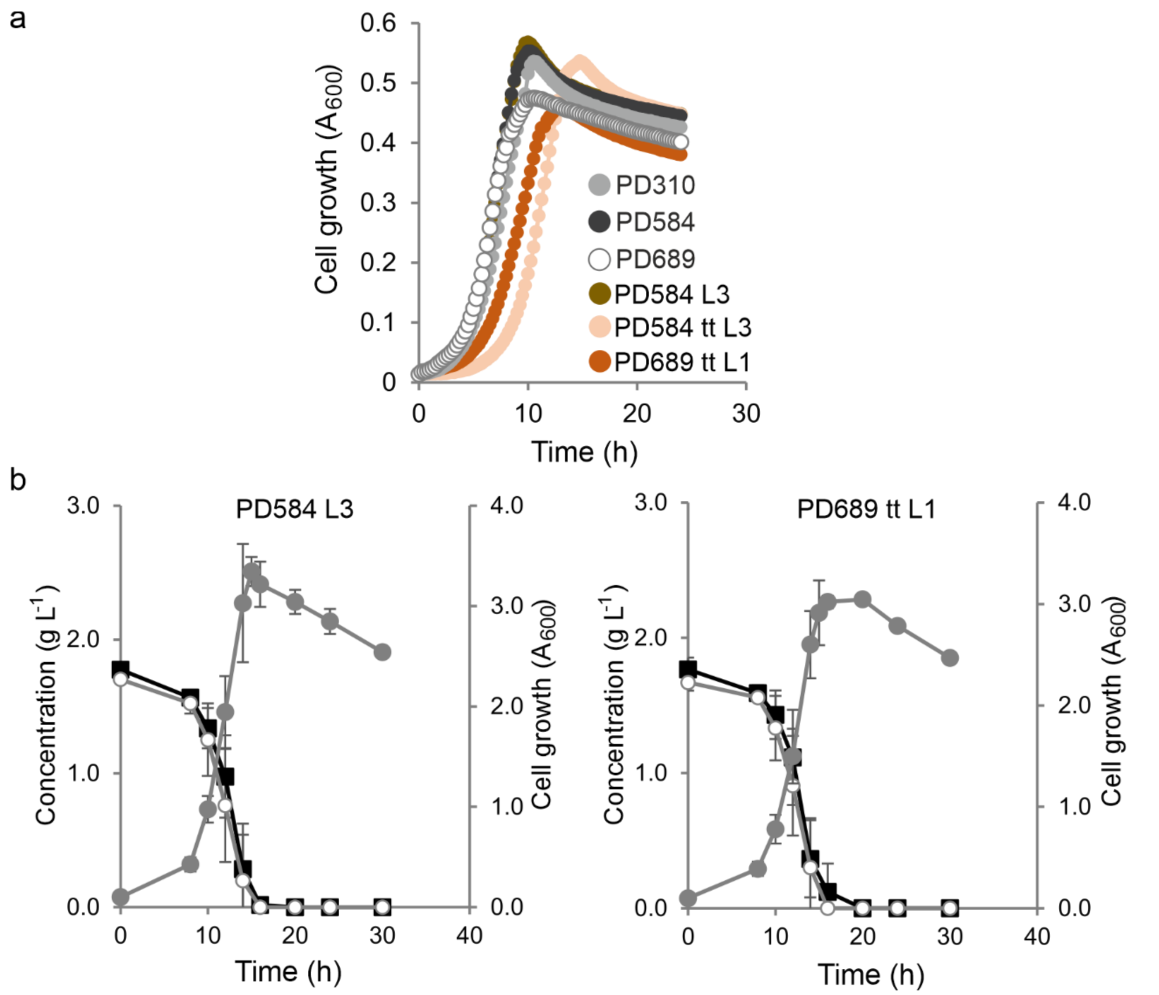

Supplementary Figure 7. Growth of engineered and evolved *Pseudomonas putida* strains on glucose (a) or on the mixture of glucose and xylose (b). (**a**)Growth of PD310, PD584, PD689, PD584 L3, PD584 tt L3, and PD689 tt in M9 medium with 2 g L^{-1} D-glucose in 48-well microplate. Data are shown as means from six (PD584 L3, PD584 tt L3, PD689 tt L1) or three (controls PD310, PD584, PD689) biological replicates. Error bars are omitted for clarity. (**b**) Shake flask cultures of *P. putida* PD584 L3 and PD689 tt L1 in M9 minimal medium with xylose and glucose (2 g L^{-1} each). Cell growth, dark grey circles; xylose, black squares; glucose, white circles. Data points represent means ± standard deviations calculated from three (n=3) biological replicates. Source data are provided as a Source Data file.

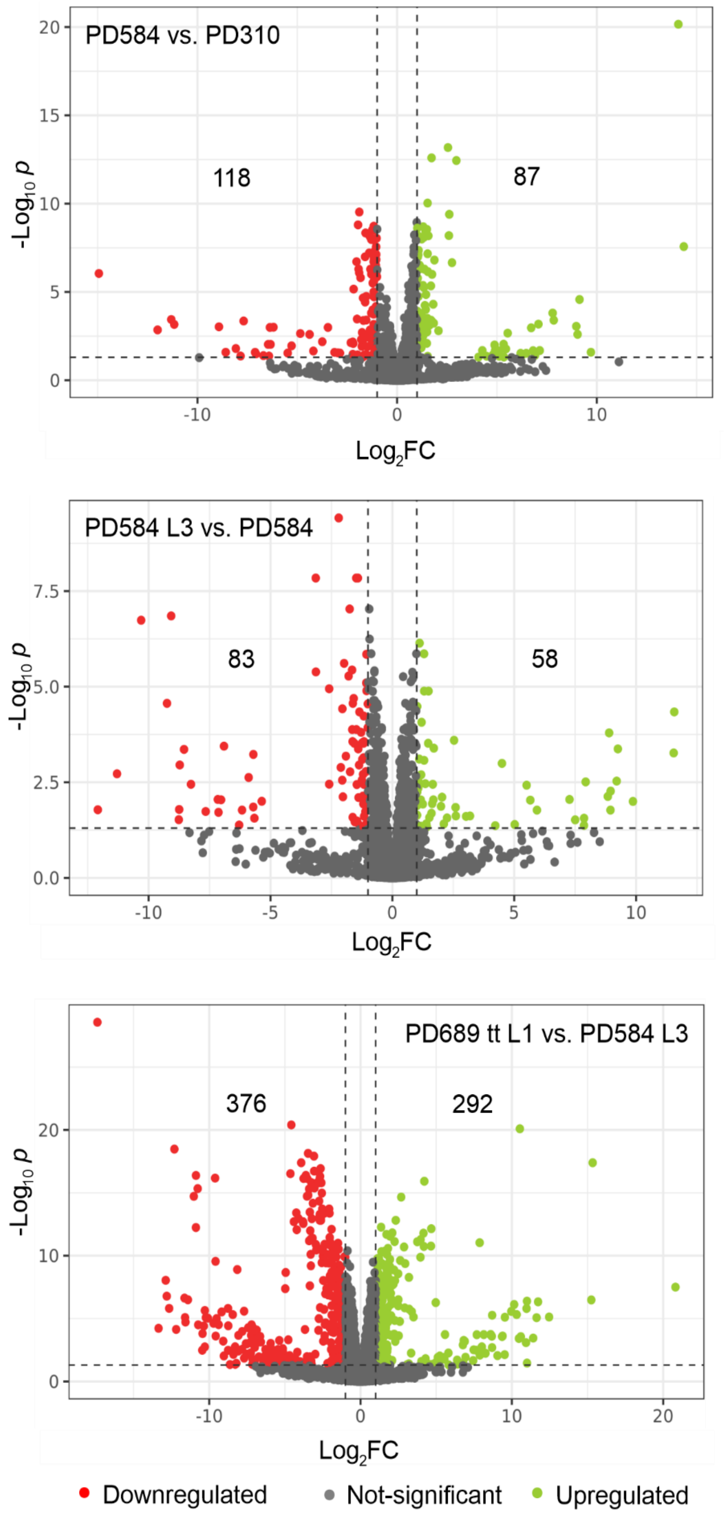

Supplementary Figure 8. Volcano plots depicting differentially abundant proteins in strain PD584 compared to PD310, PD584 L3 compared to PD584, and PD689 tt L1 compared to PD584 L3. The strains were grown in M9 minimal salt medium with 2 g L^{-1} and harvested in the mid-exponential phase. The biomass was further processed as described in Supplementary Method 9. Significantly downregulated proteins ($p < 0.05$, log_2 fold change <-1.0) are shown as red dots, significantly upregulated proteins ($p < 0.05$, log_2 fold change >1.0) are shown as green dots. Each point in the plot represents an individual protein. Source data are provided as a Source Data file.

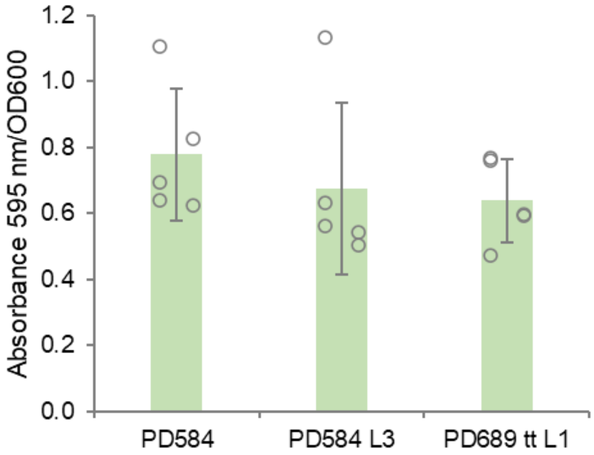

Supplementary Figure 9. Biofilm formation of three *P. putida* strains grown on 2 g L^{-1} xylose in a 96 microtiter plate, assessed by crystal violet staining. Columns represent means ± standard deviations calculated from five (n=5) biological replicates. The data show no significance of the difference between the two means of PD584 vs. PD584 L3 and PD584 vs. PD689 tt L1 ($p > 0.05$ in both cases as calculated using two-tailed Student *t* test, *p* values = 0.50 and 0.23). Source data are provided as a Source Data file.

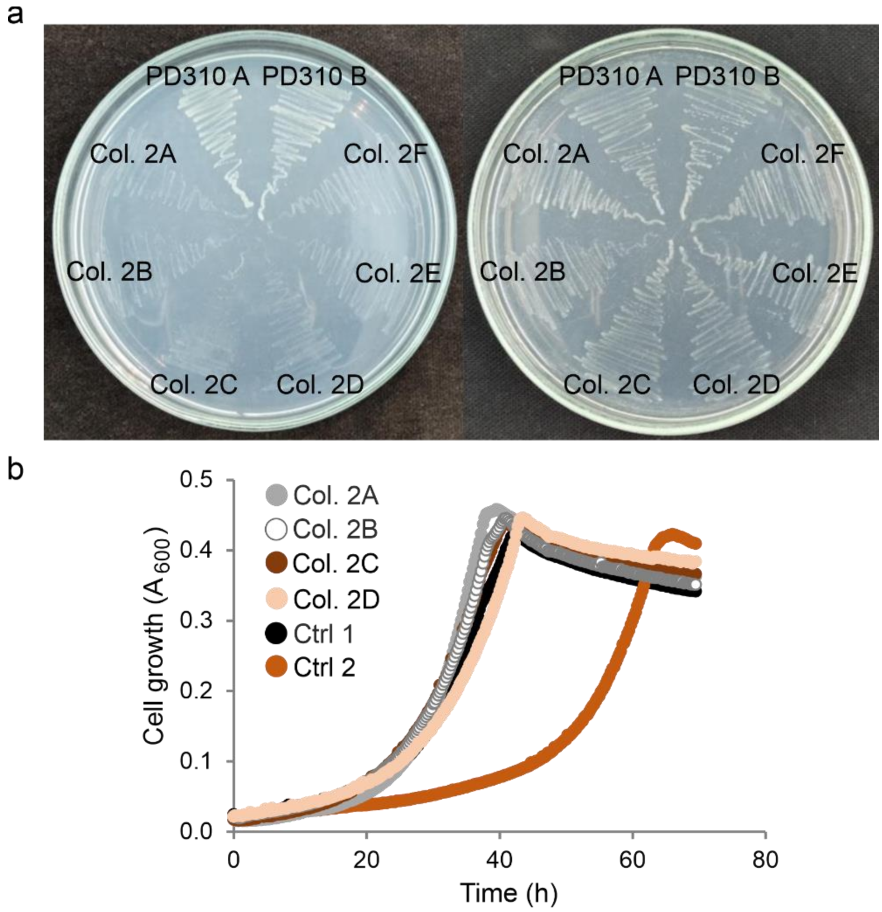

Supplementary Figure 10. Growth of several clones of freshly prepared strain *P. putida* EM42 Δ*gcd* pSEVA2213_*xylABE* (without the multiplication of the ~118 kbp segment including *tal* gene in its chromosome) and PD310 control (with multiplication) on M9 minimal salts agar with 2 g L^{-1} xylose (a) or in 48-well microplate with M9 minimal salts medium with 2 g L^{-1} xylose (b). (**a**) shows a plate with freshly streaked clones Col. 2A – Col. 2F (left) and a plate with re-streaked clones (right). (**b**) shows the growth of re-streaked clones and controls: PD310 (Ctrl 1) and the parental strain EM42 Δ*gcd* pSEVA2213_*xylABE* (Ctrl 2). Data in (**b**) are shown as mean ± standard deviation from six (n=6) biological replicates. Error bars are omitted for clarity. Source data are provided as a Source Data file.

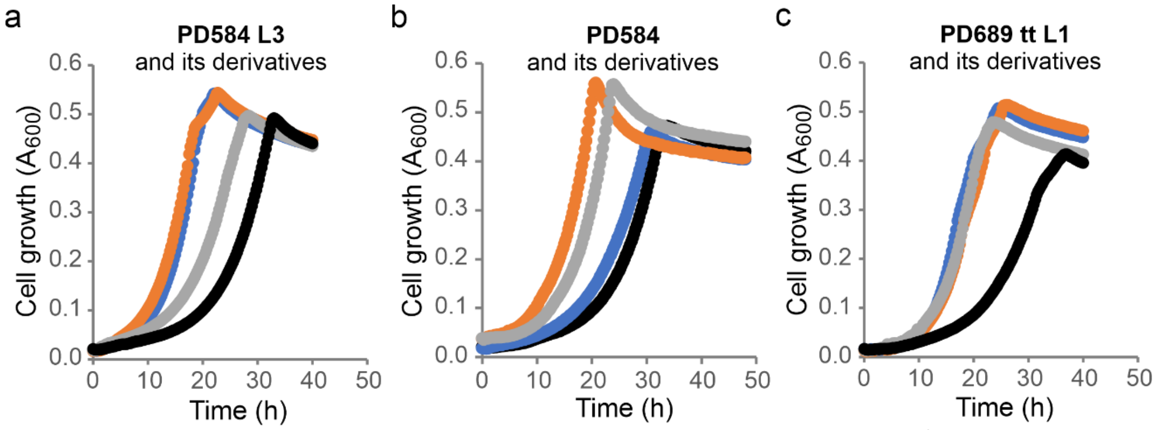

Supplementary Figure 11. Microplate growth assays on xylose (2 g L^{-1}) with PD584 L3 (a), PD584 (b), and PD689 tt L1 (c) bearing re-implanted pSEVA2213_*xylABE* plasmid. (**a**) PD584 L3 was used either intact (orange symbols) or its plasmid pSEVA2213_*xylABE* containing the duplication was removed and the strain was transformed with the same plasmid (blue symbols) or the original (flawless) pSEVA2213_*xylABE* plasmid (grey symbols). Strain PD584 was used as a control (black symbols). (**b**) PD584 was either used intact (black symbols) or its flawless plasmid pSEVA2213_*xylABE* was removed and the strain was transformed with the same plasmid (blue symbols) or with the mutated plasmid from PD584 L3 (grey symbols). PD584 L3 was used as a control (orange symbols). (**c**) PD689 tt L1 strain was used either intact (orange symbols) or its flawless pSEVA2213_*xylABE* plasmid was removed and the strain was transformed with the mutated plasmid from PD584 L3 (blue symbols) or with the original flawless pSEVA2213_*xylABE* plasmid (grey symbols). Strain PD584 was used as a control (black symbols). Data are shown as mean from six (n=6) biological replicates. Error bars are omitted for clarity. Source data are provided as a Source Data file.

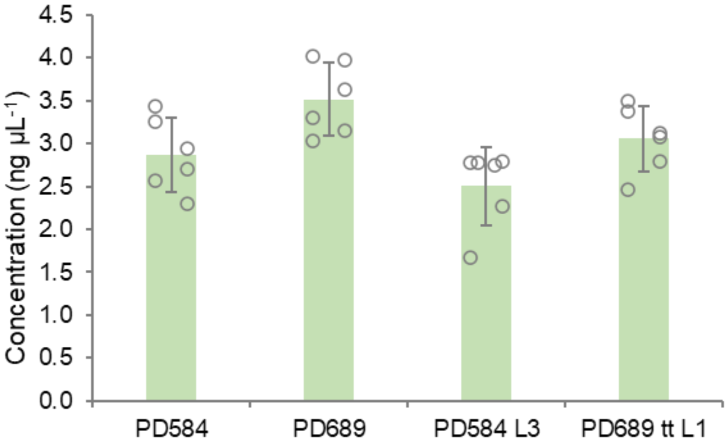

Supplementary Figure 12. The concentration of pSEVA2213_*xylABE* plasmid DNA determined by the Qubit fluorometric quantification after isolation from the selected *P. putida* strains. Overnight cultures in LB were used for inoculation of 25 mL of fresh LB medium in 250 mL Erlenmeyer flask. Cells were cultivated at 30°C at 200 rpm (NB-205, N-BIOTEK) until OD_{600} of 0.5-0.6 and 5 mL of cell suspension corresponding to OD_{600} of 0.5 was centrifuged (2,000 g, 10 min). Plasmid DNA was isolated using the GeneJET Plasmid Miniprep Kit (Thermo Fisher Scientific) according to the manufacturer's instructions. DNA concentration was measured using QubitTM Fluorometer and QubitTM 1X dsDNA High Sensitivity Assay Kit (Thermo Fisher Scientific) according to the manufacturer's instructions. Columns represent means ± standard deviations calculated from six (n=6) biological replicates. The data show no significance of the difference between the two means of PD584 (parental strain) vs. PD584 L3 (strain after adaptive evolution) and PD689 (parental strain) vs. PD689 tt L1 (strain after adaptive evolution) ($p > 0.05$ in both cases as calculated using two-tailed Student t test, p values = 0.19 and 0.08). Source data are provided as a Source Data file.

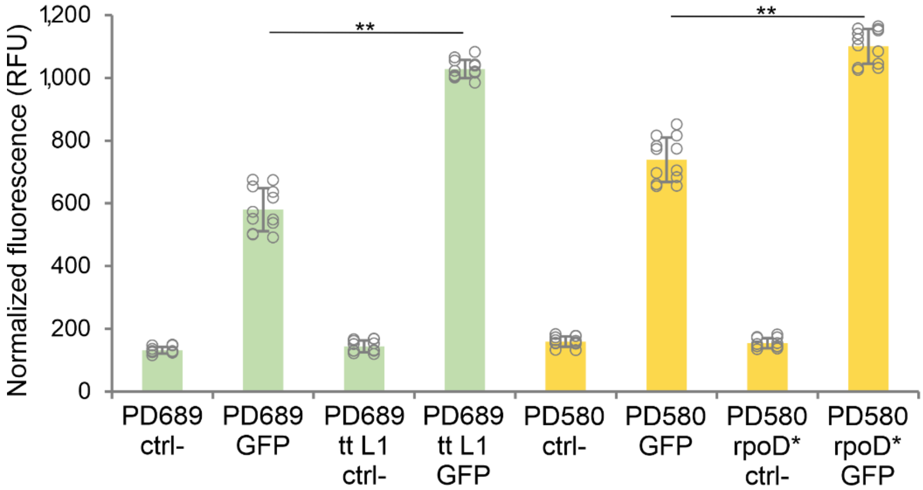

Supplementary Figure 13. Fluorescence of green fluorescent protein (GFP) detected in cell cultures of engineered *P. putida* strains. Strains PD689, PD689 tt L1, PD580, and PD580 *rpoD** (*indicates the Ser552Pro mutation in the *rpoD* gene) with the pSEVA2213_*xylABE* plasmid were used as negative controls (ctrl-) for GFP fluorescence. The same strains carrying pSEVA2213_*gfp* plasmid (*gfp* gene is under the control of the EM7 promoter) instead of pSEVA2213_*xylABE*, are designated in the figure as PD689 GFP, PD689 tt L1 GFP, PD580 GFP, and PD580 rpoD* GFP. Cells from overnight culture were inoculated into the wells of 48-well microplate with 600 µL of LB medium and kanamycin. Optical density (A_{600}) and GFP fluorescence (485/510 nm, bottom reading, manual gain 60) in cell cultures were measured for 6.5 h (mid-exponential phase) using Infinite M Plex plate reader (Tecan). Fluorescence in relative fluorescence units (RFU) detected after 6.5 h was normalized to A_{600} values of each strain. Columns represent means ± standard deviations calculated from twelve (n=12) biological replicates from two independent experiments (each of six biological replicates). Asterisks denote the significance of the difference between the two means at $p < 0.01$ calculated using two-tailed Student t test (p values = 2.17×10^{-12} and 5.11×10^{-12}). Source data are provided as a Source Data file.

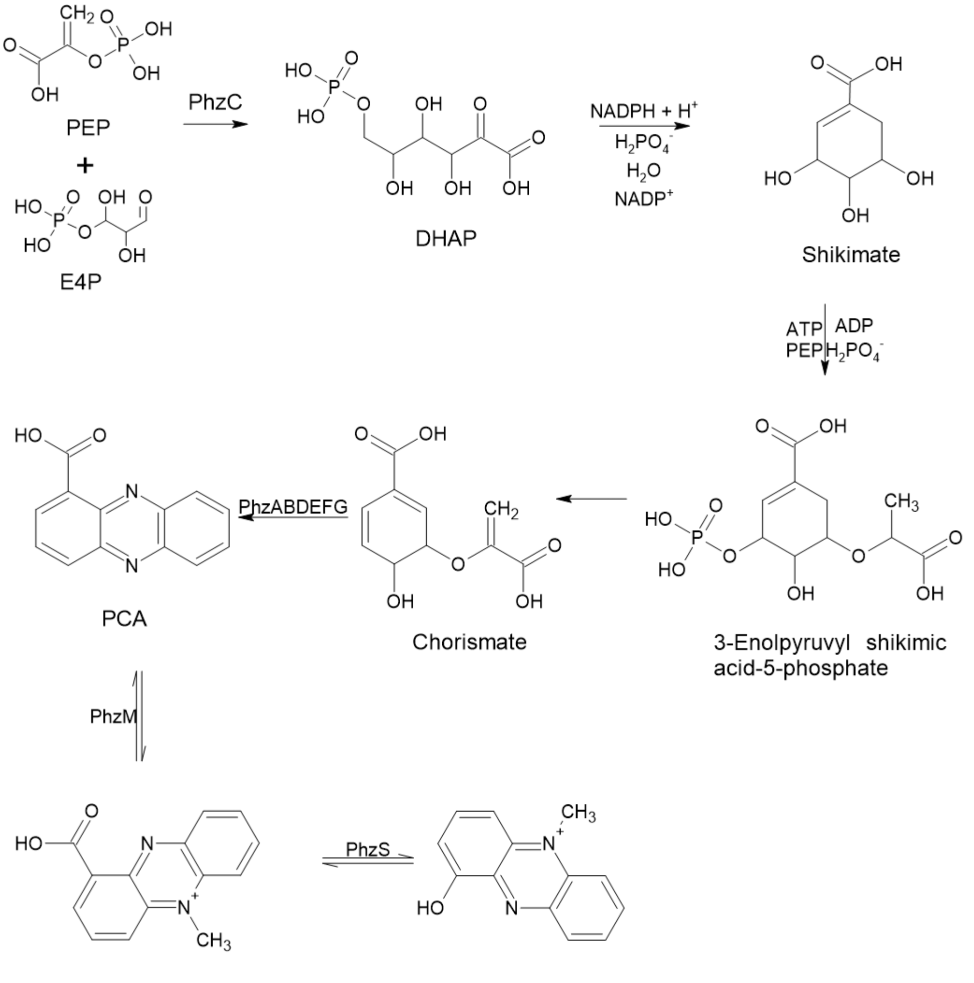

Supplementary Figure 14. Simplified schematic illustration of the phenazine biosynthetic pathway. Abbreviations (metabolites): DHAP, dihydroxyacetone phosphate; E4P, erythrose-4-phosphate; PCA, phenazine-1-carboxylic acid; PEP, phosphoenolpyruvate; (enzymes) PhzC, probable phospho-2-dehydro-3-deoxyheptonate aldolase; PhzG, dihydrophenazinedicarboxylate synthase; PhzF, trans-2,3-dihydro-3-hydroxyanthranilate isomerase; PhzM, phenazine-1-carboxylate N-methyltransferase; PhzS, 5-methylphenazine-1-carboxylate 1-monooxygenase; PhzABDE, phenazine biosynthesis protein.

Supplementary references

1. Bradford, M. M. A rapid and sensitive method for the quantitation of microgram quantities of protein utilizing the principle of protein-dye binding. *Anal. Biochem.* **72**, 248–254 (1976).
2. Dvořák, P. & de Lorenzo, V. Refactoring the upper sugar metabolism of *Pseudomonas putida* for co-utilization of cellobiose, xylose, and glucose. *Metab. Eng.* **48**, 94–108 (2018).
3. Stephenson, M. P., Jackson, F. A. & Dawes, E. A. Y. Further observations on carbohydrate metabolism and its regulation in *Azotobacter beijerinckii*. *Microbiology* **109**, 89–96 (1978).
4. Sánchez-Pascuala, A., de Lorenzo, V. & Nikel, P. I. Refactoring the Embden–Meyerhof–Parnas pathway as a whole of portable GlucoBricks for implantation of glycolytic modules in Gram-negative bacteria. *ACS Synth. Biol.* **6**, 793–805 (2017).
5. Zhu, X. *et al.* The CRISPR/Cas9-facilitated multiplex pathway optimization (CFPO) technique and its application to improve the *Escherichia coli* xylose utilization pathway. *Metab. Eng.* **43**, 37–45 (2017).
6. Sobota, J. M. & Imlay, J. A. Iron enzyme ribulose-5-phosphate 3-epimerase in *Escherichia coli* is rapidly damaged by hydrogen peroxide but can be protected by manganese. *Proc. Natl. Acad. Sci. U. S. A.* **108**, 5402–5407 (2011).
7. Martínez-García, E. & de Lorenzo, V. Transposon-based and plasmid-based genetic tools for editing genomes of gram-negative bacteria. *Methods Mol. Biol. Clifton NJ* **813**, 267–283 (2012).
8. Geu-Flores, F., Nour-Eldin, H. H., Nielsen, M. T. & Halkier, B. A. USER fusion: a rapid and efficient method for simultaneous fusion and cloning of multiple PCR products. *Nucleic Acids Res.* **35**, e55 (2007).
9. Volke, D. C., Friis, L., Wirth, N. T., Turlin, J. & Nikel, P. I. Synthetic control of plasmid replication enables target- and self-curing of vectors and expedites genome engineering of *Pseudomonas putida*. *Metab. Eng. Commun.* **10**, e00126 (2020).
10. Watson, J. F. & García-Nafría, J. In vivo DNA assembly using common laboratory bacteria: A re-emerging tool to simplify molecular cloning. *J. Biol. Chem.* **294**, 15271–15281 (2019).
11. Bator, I., Wittgens, A., Rosenau, F., Tiso, T. & Blank, L. M. Comparison of three xylose pathways in *Pseudomonas putida* KT2440 for the synthesis of valuable products. *Front. Bioeng. Biotechnol.* **7**, 480 (2020).
12. Schmitz, S., Nies, S., Wierckx, N., Blank, L. M. & Rosenbaum, M. A. Engineering mediator-based electroactivity in the obligate aerobic bacterium *Pseudomonas putida* KT2440. *Front. Microbiol.* **6**, (2015).
13. Bujdoš, D. *et al.* Engineering of *Pseudomonas putida* for accelerated co-utilization of glucose and cellobiose yields aerobic overproduction of pyruvate explained by an upgraded metabolic model. *Metab. Eng.* **75**, 29–46 (2023).
14. Long, C. P. & Antoniewicz, M. R. Metabolic flux analysis of *Escherichia coli* knockouts: lessons from the Keio collection and future outlook. *Curr. Opin. Biotechnol.* **28C**, 127–133 (2014).
15. Swain, P. S. *et al.* Inferring time derivatives including cell growth rates using Gaussian processes. *Nat. Commun.* **7**, 13766 (2016).
16. Fišarová, L. *et al. Staphylococcus epidermidis* phages transduce antimicrobial resistance plasmids and mobilize chromosomal islands. *mSphere* **6**, e00223-21 (2021).
17. Bolger, A. M., Lohse, M. & Usadel, B. Trimmomatic: a flexible trimmer for Illumina sequence data. *Bioinformatics* **30**, 2114–2120 (2014).
18. Wick, R. R., Judd, L. M., Gorrie, C. L. & Holt, K. E. Unicycler: Resolving bacterial genome assemblies from short and long sequencing reads. *PLOS Comput. Biol.* **13**, e1005595 (2017).

19. Walker, B. J. *et al.* Pilon: An integrated tool for comprehensive microbial variant detection and genome assembly improvement. *PLOS ONE* **9**, e112963 (2014).
20. Lin, Y. *et al.* Assembly of long error-prone reads using de Bruijn graphs. *Proc. Natl. Acad. Sci.* **113**, E8396–E8405 (2016).
21. Seemann, T. Prokka: rapid prokaryotic genome annotation. *Bioinformatics* **30**, 2068–2069 (2014).
22. Tatusova, T. *et al.* NCBI prokaryotic genome annotation pipeline. *Nucleic Acids Res.* **44**, 6614–6624 (2016).
23. Wiśniewski, J. R., Ostasiewicz, P. & Mann, M. High recovery FASP applied to the proteomic analysis of microdissected formalin fixed paraffin embedded cancer tissues retrieves known colon cancer markers. *J. Proteome Res.* **10**, 3040–3049 (2011).
24. Yeung, Y.-G., Nieves, E., Angeletti, R. H. & Stanley, E. R. Removal of detergents from protein digests for mass spectrometry analysis. *Anal. Biochem.* **382**, 135–137 (2008).
25. Stejskal, K., Potěšil, D. & Zdráhal, Z. Suppression of peptide sample losses in autosampler vials. *J. Proteome Res.* **12**, 3057–3062 (2013).
26. Demichev, V., Messner, C. B., Vernardis, S. I., Lilley, K. S. & Ralser, M. DIA-NN: Neural networks and interference correction enable deep proteome coverage in high throughput. *Nat. Methods* **17**, 41–44 (2020).
27. Nogales, J. *et al.* High-quality genome-scale metabolic modelling of *Pseudomonas putida* highlights its broad metabolic capabilities. *Environ. Microbiol.* **22**, 255–269 (2020).
28. Elmore, J. R. *et al.* Engineered *Pseudomonas putida* simultaneously catabolizes five major components of corn stover lignocellulose: Glucose, xylose, arabinose, *p*-coumaric acid, and acetic acid. *Metab. Eng.* **62**, 62–71 (2020).
29. Valdivia, M., Galan, J. L., Laffarga, J. & Ramos, J.-L. Biofuels 2020: Biorefineries based on lignocellulosic materials. *Microb. Biotechnol.* **9**, 585–594 (2016).
30. Jin, D. J., Zhou, Y. N., Shaw, G. & Ji, X. Structure and function of RapA: A bacterial Swi2/Snf2 protein required for RNA polymerase recycling in transcription. *Biochim. Biophys. Acta* **1809**, 470–475 (2011).
31. Stellmacher, L. *et al.* Novel mode of inhibition by D-tagatose 6-phosphate through a Heyns rearrangement in the active site of transaldolase B variants. *Acta Crystallogr. Sect. Struct. Biol.* **72**, 467–476 (2016).
32. Grant, S. G., Jessee, J., Bloom, F. R. & Hanahan, D. Differential plasmid rescue from transgenic mouse DNAs into *Escherichia coli* methylation-restriction mutants. *Proc. Natl. Acad. Sci. U. S. A.* **87**, 4645–4649 (1990).
33. Manoil, C. & Beckwith, J. TnphoA: a transposon probe for protein export signals. *Proc. Natl. Acad. Sci. U. S. A.* **82**, 8129–8133 (1985).
34. Herrero, M., de Lorenzo, V. & Timmis, K. N. Transposon vectors containing non-antibiotic resistance selection markers for cloning and stable chromosomal insertion of foreign genes in gram-negative bacteria. *J. Bacteriol.* **172**, 6557–6567 (1990).
35. Boyer, H. W. & Roulland-Dussoix, D. A complementation analysis of the restriction and modification of DNA in *Escherichia coli*. *J. Mol. Biol.* **41**, 459–472 (1969).
