# Supplementary material for: Synthetically-primed adaptation of Pseudomonas putida to a non-native substrate D-xylose
Source: Nat Commun. 2024 Mar 26;15:2666. doi: 10.1038/s41467-024-46812-9 (PMC10965963; doi:10.1038/s41467-024-46812-9)
Supplement: Supplementary file 3 — Description of Additional Supplementary Files [file 41467_2024_46812_MOESM3_ESM.pdf]

Description of Additional Supplementary Files

File Name: Supplementary Data 1
Description: Summary of fluxes through NAD(P)H producing reactions from MFA and FBA.

File Name: Supplementary Data 2
Description: Analysis of proteomes of *P. putida* strains grown on xylose. The table contains original data from three proteome comparisons: PD584 vs. PD310, PD584 L3 vs. PD584, and PD689 tt L1 vs. PD584 L3.

File Name: Supplementary Data 3
Description: Changes identified in the genome of sequenced strain PD584 L3 after adaptive laboratory evolution when compared with the reference strain PD584.

File Name: Supplementary Data 4
Description: Changes identified in the genome of sequenced strain PD689 tt L1 after adaptive laboratory evolution when compared with the reference strain PD689.

File Name: Supplementary Data 5
Description: Table of annotated genes positioned in the ~118 kbp multiplied region identified after the whole-genome sequencing of PD584 L3, PD584, and PD310 strains.

File Name: Supplementary Data 6
Description: Plasmids used in this study.

File Name: Supplementary Data 7
Description: Quantities of selected proteins (absolute quantification) encoded by the genes on the multiplied region in the chromosome of strains PD310, PD584, and PD584 L3 compared to the reference strain PD689 tt L1 without the multiplication.

File Name: Supplementary Data 8
Description: Oligonucleotide primers used in this study.

File Name: Supplementary Data 9
Description: Nucleotide sequences of synthetic expression cassettes used in this study.
