# Supplementary material for: Synthetically-primed adaptation of Pseudomonas putida to a non-native substrate D-xylose
Source: Nat Commun. 2024 Mar 26;15:2666. doi: 10.1038/s41467-024-46812-9 (PMC10965963; doi:10.1038/s41467-024-46812-9)
Supplement: Supplementary file 13 — Reporting Summary [file 41467_2024_46812_MOESM13_ESM.pdf]

nature portfolio

Corresponding author(s): Pavel Dvorak

Last updated by author(s): Mar 8, 2024

Reporting Summary

Nature Portfolio wishes to improve the reproducibility of the work that we publish. This form provides structure for consistency and transparency in reporting. For further information on Nature Portfolio policies, see our Editorial Policies and the Editorial Policy Checklist.

Statistics

For all statistical analyses, confirm that the following items are present in the figure legend, table legend, main text, or Methods section.

n/a	Confirmed	
☐	☒	The exact sample size (*n*) for each experimental group/condition, given as a discrete number and unit of measurement
☐	☒	A statement on whether measurements were taken from distinct samples or whether the same sample was measured repeatedly
☐	☒	The statistical test(s) used AND whether they are one- or two-sided<br>*Only common tests should be described solely by name; describe more complex techniques in the Methods section.*
☒	☐	A description of all covariates tested
☐	☒	A description of any assumptions or corrections, such as tests of normality and adjustment for multiple comparisons
☐	☒	A full description of the statistical parameters including central tendency (e.g. means) or other basic estimates (e.g. regression coefficient) AND variation (e.g. standard deviation) or associated estimates of uncertainty (e.g. confidence intervals)
☐	☒	For null hypothesis testing, the test statistic (e.g. *F*, *t*, *r*) with confidence intervals, effect sizes, degrees of freedom and *P* value noted<br>*Give P values as exact values whenever suitable.*
☒	☐	For Bayesian analysis, information on the choice of priors and Markov chain Monte Carlo settings
☒	☐	For hierarchical and complex designs, identification of the appropriate level for tests and full reporting of outcomes
☒	☐	Estimates of effect sizes (e.g. Cohen's *d*, Pearson's *r*), indicating how they were calculated

Our web collection on statistics for biologists contains articles on many of the points above.

Software and code

Policy information about availability of computer code

Data collection	Data from bacterial microplate cultures were collected using I-Control v. 2.0 (Tecan) and Microsoft Office Excell 365. To predict flux distributions by flux balance analysis either COBRApy library (Ebrahim at al., 2013) or MATLAB COBRA toolbox v3.0 (Heirendt et al., 2019) was used. Oligonucleotide primers were designed using AMUSER 1.0 software (Genee et al. 2015). Synthetic ribosome binding sites were designed by RBS Calculator v2.1 (De Novo DNA).<br>Code availability<br>Two modified genome-scale metabolic models of P. putida used in this study are available from GitHub [https://github.com/DalimilBujdos/P_putida_xylose_metabolism].
Data analysis	Plate reader data were plotted and analyzed using the deODorizer program v1.0. (Swain et al., 2016).<br>Next-generation sequencing data were analyzed using Geneious Prime v2022.2.2 (Biomatters). Illumina reads were trimmed and filtered using Trimmomatic v0.38.1. Complete bacterial genome sequences were obtained using a hybrid assembly with Unicycler v0.4.8. The resulting assembly was polished with Pilon v1.24. Assembly of Nanopore-only reads was performed using Flye v.2.9.1 and Medaka consensus pipeline v1.7.2 (Oxford Nanopore). Prokka v1.14.6 was used to annotate gene products for in-house proteomic analysis. Sequence alignments were carried out in Benchling.<br>The genomes were annotated using the NCBI Prokaryotic Genome Annotation Pipeline.<br>Strength of selected ribosom-binding sites was evaluated using RBS Calculator v2.1 (De Novo DNA).<br>Data from shake flask and microplate cultures or enzyme activity measurements were processed using Microsoft Office Excel 365<br>Data from labeling experiments were corrected for unlabeled biomass and natural abundance of heavy isotopes using the software iMS2FLUX v7.2.1 (Poskar et al., 2012).<br>Metabolic flux analysis was performed using the Matlab-based tool INCA.

DIA proteomic data were processed in DIA-NN v1.8, protein intensities were further processed using the software container environment (https://github.com/OmicsWorkflows) v4.1.3a. Duplicities were removed in Microsoft Office Excel 365. Gene identifiers were coupled to UniProt identifiers using the ID mapping tool available at uniprot.org. Data were visualized in Escher (escher.github.io).
Capillary IC-MS data were corrected for the natural abundance of heavy isotopes using IsoCorr.

For manuscripts utilizing custom algorithms or software that are central to the research but not yet described in published literature, software must be made available to editors and reviewers. We strongly encourage code deposition in a community repository (e.g. GitHub). See the Nature Portfolio guidelines for submitting code & software for further information.

Data

Policy information about availability of data

All manuscripts must include a data availability statement. This statement should provide the following information, where applicable:

- Accession codes, unique identifiers, or web links for publicly available datasets
- A description of any restrictions on data availability
- For clinical datasets or third party data, please ensure that the statement adheres to our policy

Supplementary and source data are provided with this paper as Supplementary Information, nine Supplementary Data files and Source Data file. All sequencing data and assembled whole-genome sequences were deposited under NCBI BioProject PRJNA914626 [https://www.ncbi.nlm.nih.gov/bioproject/PRJNA914626]. The whole-genome sequences and raw sequencing data have been deposited in the GenBank database and SRA database (NCBI), respectively, under accession numbers summarized in Supplementary Table 1. The mass spectrometry proteomics data have been deposited to the ProteomeXchange Consortium via the PRIDE partner repository with the dataset identifier PXD047537 [https://www.ebi.ac.uk/pride/archive/projects/PXD047537]. The raw gas chromatography-mass spectrometry and ion chromatography-mass spectrometry data used for the metabolic flux analysis in this study have been deposited in the Zenodo repository under DOI:10.5281/zenodo.10732391 [https://zenodo.org/records/10732391].

Research involving human participants, their data, or biological material

Policy information about studies with human participants or human data. See also policy information about sex, gender (identity/presentation), and sexual orientation and race, ethnicity and racism.

Reporting on sex and gender	N/A
Reporting on race, ethnicity, or other socially relevant groupings	N/A
Population characteristics	N/A
Recruitment	N/A
Ethics oversight	N/A

Note that full information on the approval of the study protocol must also be provided in the manuscript.

Field-specific reporting

Please select the one below that is the best fit for your research. If you are not sure, read the appropriate sections before making your selection.

[x] Life sciences [ ] Behavioural & social sciences [ ] Ecological, evolutionary & environmental sciences

For a reference copy of the document with all sections, see nature.com/documents/nr-reporting-summary-flat.pdf

Life sciences study design

All studies must disclose on these points even when the disclosure is negative.

Sample size	All shake flask cell cultures and cultures in microplates as well as measurements of enzymatic activities were performed in at least three (n=3) biological replicates. Key experiments were performed with six (n=6) or more biological replicates in single experiment or in two independent experiments. 13C labeling experiment with labeled xylose was performed in biological duplicate (n=2). Proteomic analyses were performed on cells from cultures run in four (n=4) biological replicates. Adaptive laboratory evolution (ALE) was performed with single cell culture (n=1) of each of the employed strains except for PD310 control strain (here n=2). Screening microplate cultures after ALE were run in single biological replicate per mutant (n=1). The sample size of at least n=3 for cell cultures, enzyme activity assays, or proteomic analyses and sample size of n=2 for labeling experiment and n=1 for screening assays were determined by experience in this research area, which has been proven consistent and sufficient to support the conclusions in the research area.
Data exclusions	No data were excluded.
Replication	All key culture experiments and enzyme activity measurements in our study were performed at least in biological triplicate. We have run multiple culture experiments with the key mutant strains presented in this study (particularly PD310, PD584, PD584 L3, PD689 tt L1, PD855) and the results are reproducible.

Randomization	Bacterial cultures in shake-flask or microplate format were randomly assigned to different strains. Flasks and tubes with overnight cultures were randomly distributed in shaking incubator. Different parts of a microplate were assigned for enzyme activity measurements.
Blinding	The researchers performing analyses of cell growth and enzyme activities were blinded for strain and enzyme information, respectively.

Reporting for specific materials, systems and methods

We require information from authors about some types of materials, experimental systems and methods used in many studies. Here, indicate whether each material, system or method listed is relevant to your study. If you are not sure if a list item applies to your research, read the appropriate section before selecting a response.

Materials & experimental systems

n/a	Involved in the study
☒	☐ Antibodies
☒	☐ Eukaryotic cell lines
☒	☐ Palaeontology and archaeology
☒	☐ Animals and other organisms
☒	☐ Clinical data
☒	☐ Dual use research of concern
☒	☐ Plants

Methods

n/a	Involved in the study
☒	☐ ChIP-seq
☒	☐ Flow cytometry
☒	☐ MRI-based neuroimaging
